# Functional Characterization of *4*′*OMT* and *7OMT* Genes in BIA Biosynthesis

**DOI:** 10.3389/fpls.2016.00098

**Published:** 2016-02-16

**Authors:** Tugba Gurkok, Esma Ozhuner, Iskender Parmaksiz, Sebahattin Özcan, Mine Turktas, Arif İpek, Ibrahim Demirtas, Sezer Okay, Turgay Unver

**Affiliations:** ^1^Eldivan SHMYO, Department of Anesthesia, Cankiri Karatekin UniversityCankiri, Turkey; ^2^Department of Biology, Faculty of Science, Cankiri Karatekin UniversityCankiri, Turkey; ^3^Department of Molecular Biology and Genetics, Faculty of Science, Gaziosmanpasa UniversityTokat, Turkey; ^4^Department of Field Crops, Faculty of Agriculture, Ankara UniversityAnkara, Turkey; ^5^Department of Chemistry, Faculty of Science, Cankiri Karatekin UniversityCankiri, Turkey

**Keywords:** metabolic engineering, morphine, noscapine, overexpression, stem tissue, VIGS

## Abstract

Alkaloids are diverse group of secondary metabolites generally found in plants. Opium poppy (*Papaver somniferum* L.), the only commercial source of morphinan alkaloids, has been used as a medicinal plant since ancient times. It produces benzylisoquinoline alkaloids (BIA) including the narcotic analgesic morphine, the muscle relaxant papaverine, and the anti-cancer agent noscapine. Though BIAs play crucial roles in many biological mechanisms their steps in biosynthesis and the responsible genes remain to be revealed. In this study, expressions of *3-hydroxy-N-methylcoclaurine 4*′–*methyltransferase* (*4*′*OMT*) and *reticuline 7-O-methyltransferase* (*7OMT*) genes were subjected to manipulation to functionally characterize their roles in BIA biosynthesis. Measurements of alkaloid accumulation were performed in leaf, stem, and capsule tissues accordingly. Suppression of *4*′*OMT* expression caused reduction in the total alkaloid content in stem tissue whereas total alkaloid content was significantly induced in the capsule. Silencing of the *7OMT* gene also caused repression in total alkaloid content in the stem. On the other hand, over-expression of *4*′*OMT* and *7OMT* resulted in higher morphine accumulation in the stem but suppressed amount in the capsule. Moreover, differential expression in several BIA synthesis genes (*CNMT, TYDC, 6OMT, SAT, COR, 4*′*OMT*, and *7OMT*) were observed upon manipulation of *4*′*OMT* and *7OMT* expression. Upon silencing and overexpression applications, tissue specific effects of these genes were identified. Manipulation of *4*′*OMT* and *7OMT* genes caused differentiated accumulation of BIAs including morphine and noscapine in capsule and stem tissues.

## Introduction

Most plants synthesize different kinds of natural products possessing commercial value such as secondary metabolites in response to various environmental or developmental factors. Alkaloids, as a member of secondary metabolites, classified into several groups including benzylisoquinoline alkaloids (BIA), commonly used for pharmaceutical purposes (Winzer et al., 2012). The opium poppy (*Papaver somniferum* L.), belongs to the Papaveraceae family and has been used as a medicine or drug for a long time (Schiff, 2002). It produces a number of BIAs including the narcotic-analgesic morphine, the cough suppressant codeine, the muscle relaxant papaverine, and the anti-microbial sanguinarine (Allen et al., 2004; Desgagné-Penix et al., 2010; Gurkok et al., 2015). Since BIA takes roles in many biological functions both in plants and animals, its biosynthesis and regulation mechanism has been an interest for researchers.

**Figure 1:**
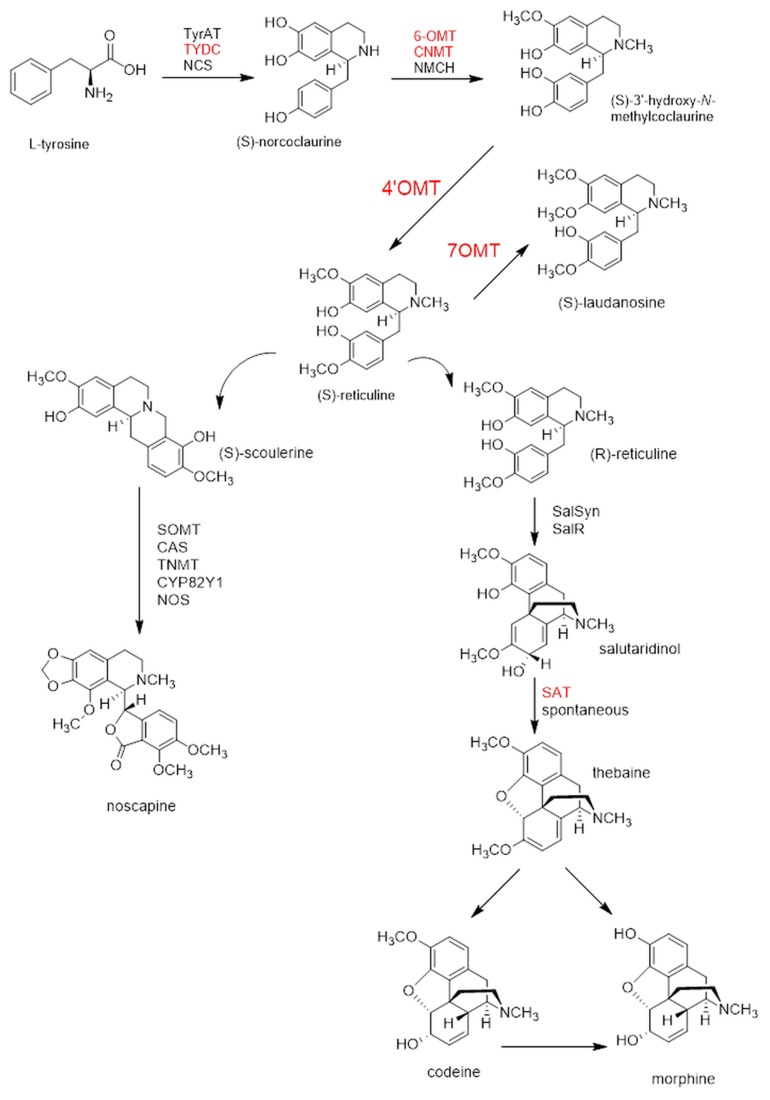
**BIA biosynthesis pathway representing the manipulated genes and several analyzed transcripts after VIGS and overexpression assays**. Both 4′*OMT* [(S)-3′-hydroxy- N-methylcoclaurine 4′-O-methyltransferase] and 7OMT (reticuline 7-O-methyltransferase) were manipulated. Transcripts that analyzed via qRT-PCR were shown in red. Long arrows point out more than one reaxion. Abbreviations: TyrAT, tyrosine aminotransferase; TYDC, tyrosine decarboxylase; NCS, norcoclaurine synthase; 6-OMT, (S)-norcoclaurine 6-O-methyltransferase; CNMT, (S)-coclaurine N-methyltransferase; NMCH, (S)-N-methylcoclaurine-3′-hydroxylase; SalSyn, salutaridine synthase; SalR, salutaridine reductase; SAT, 7(S)- salutaridinol 7-O-acetyltransferase; CODM, codeine O-demethylase; T6ODM, thebaine 6-O-demethylase; COR, codeinone reductase; SOMT, scoulerine 9-O-methyltransferase; CAS, (S)-canadine synthase; TNMT; tetrahydroprotoberberine N-methyltransferase; CYP82Y1, N-methylcanadine 1-hydroxylase; NOS, noscapine synthase.

To date several genes involved in the BIA biosynthesis in opium poppy have been cloned and characterized. The BIA biosynthesis begins with the condensation of two L-tyrosine derivatives- 4′hydroxyphenylacetaldehyde (4′HPAA) and dopamine- catalyzed by tyrosine/DOPA decarboxylase (TYDC) to generate (S)-norcoclourine (Facchini and De Luca, 1994; Lee and Facchini, 2011). Different types of BIA like protoberberine, morphinan, and others share the early common steps in the biosynthetic pathway. (S)-reticuline is the central intermediate of opium BIA ramification and its formation needs a series of enzymes including norcoclaurine synthase (NCS; Lee and Facchini, 2010), norcoclaurine 6-*O*-methyltransferase (6OMT; Morishige et al., 2000), coclaurine N-methyltransferase (CNMT; Choi et al., 2002), and 3-hydroxy-N-methylcoclaurine 4′-O-methyltransferase (4′*OMT*; Morishige et al., 2000). Although, (R,S)-reticuline7-*O*-methyltransferase (7OMT), converts (S)-reticuline to (S)-laudanine (Ounaroon et al., 2003) the morphinan branch requires the epimerization of (S)-reticuline to (R)- reticuline (De-Eknamkul and Zenk, 1992). (R)-reticuline is converted into salutaridine by salutaridine synthase (SalSyn), which is then reduced by salutaridine reductase to yield salutaridinol (Ziegler et al., 2006). The following step is the conversion of salutaridinol to salutaridinol-7-*O*-acetate via the enzyme salutaridinol-7-*O*-acetyltransferase (SAT; Grothe et al., 2001). In the last step of morphine biosynthesis, the conversion of thebaine to morphine occurs via codeine or oripavine produced by the enzymes thebaine 6-*O*-demethylase (T6ODM), codeinone reductase (COR), and codeine *O*-demethylase (Figure 1) (CODM; Unterlinner et al., 1999; Hagel and Facchini, 2010). Albeit a lot of enzymes cloned, BIA biosynthesis, and regulation has not been fully discerned yet.

Metabolic engineering is an approach that alters metabolic pathways and/or metabolite production via gene transfer technology. Biotechnological manipulation of a plant's secondary metabolism pathways, such as gene silencing or overexpression, aids in the discovery of gene functions as well as in increasing the accumulation of the high-value plant's natural products (Nagamatsu et al., 2007; Purkayastha and Dasgupta, 2009; Bedon et al., 2010). On the other hand, Virus-induced gene silencing (VIGS) is an emerging technique to study gene functions. Since plants induce homology-dependent defense mechanisms in response to viral attacks VIGS are advantageous over other silencing approaches (Hileman et al., 2005). VIGS, leading a fast and transient suppression of gene expression, involves cloning of short sequence fragments of interested targeted gene to be silenced (Unver and Budak, 2009). Tobacco rattle viruses (TRV) used as silencing vectors which have been engineered to silence target genes in intended host plants including opium poppy (Liu et al., 2002). TRV mediated VIGS can be applied in different plant tissues to silence metabolic pathway genes. Furthermore, to investigate the regulation of morphine biosynthesis, the genes responsible for the last six steps of morphine synthesis were systemically silenced via VIGS. As a result, it was observed that by the silencing of *SalSyn, SalR, T6ODM*, and *CODM* genes, morphine content was reduced whereas salutaridine, thebaine and codeine accumulation was remarkably induced (Wijekoon and Facchini, 2012). Furthermore, some genes including *4*′*OMT* and *7OMT* were silenced to enlighten the papaverine biosynthesis in papaverine rich plants (Desgagné-Penix and Facchini, 2012). Winzer et al. (2012) carried out VIGS assays to reveal the genes taking part in noscapine biosynthesis. In this study, pathway intermediate accumulation led to the observation of a new gene functioning in noscapine biosynthesis as well as a novel pathway. Additionally, Allen et al. (2004) used RNA interference (RNAi) to block codeinone reductase (COR) activity, which is the first example of metabolic engineering application on opium poppy. After suppression of COR, the common intermediate alkaloid (S)-reticuline, precursor of diverse BIA, was accumulated in transgenic plants and in this study it was suggested that there is a feedback mechanism in BIA biosynthesis.

Besides gene suppression studies, overexpression assays were also conducted for various genes in BIA biosynthesis. For example, overexpression of *COR1* caused increased morphine and codeine accumulation ~22 and 58%, respectively, in opium poppy (Larkin et al., 2007). *SAT* gene overexpression increased the total alkaloid content by about 40% (Allen et al., 2008). Noteworthy, overexpression of *NMCH* raised the total alkaloid level to 450% in opium poppy (Frick et al., 2007). The overexpression of O-methyltransferases *6OMT* and *4*′*OMT*, the upstream enzymes of BIA pathway in California poppy *Eschscholzia californica* cells, suggested a rate-limiting role for 6OMT since it caused an increase in total alkaloid content. However, little effect was found to be caused by *4*′*OMT* (Inui et al., 2007). These results indicate that unexpected outputs can be seen in BIA pathway because of the unidentified branches.

In the current study, to address the tissue specific functional roles of *4*′*OMT* and *7OMT* genes in BIA biosynthesis TRV-based VIGS, which is a useful method to silence the expression of genes in plants and transient overexpression approaches, were utilized. In order to observe the alkaloid accumulation alteration. Upon genetic manipulation in stem, capsule, and leaves of opium poppy, alkaloid levels were measured by HPLC-TOF/MS. Expression level of the genes involved in the BIA biosynthetic pathway (*CNMT, TYDC, 6OMT, SAT, COR, 4*′*OMT*, and *7OMT*) were also quantified via qRT-PCR.

## Materials and methods

### Plant material and growth conditions

*Papaver somniferum* cv Ofis 95, a morphine rich variety, seeds were provided from Toprak Mahsulleri Ofisi (TMO; Ankara, Turkey). These were potted in %25 peats, %25 perlite, and %50 soil mixtures, and then transferred to a growth chamber. Plants were kept with day/night cycles of 16/8 h at 20/24°C photoperiod and 3 month-old leaves were selected for gene cloning experiments.

### RNA isolation and cDNA synthesis

Collected tissues were ground to a fine powder in liquid nitrogen. RNA isolations were performed by TRIzol® Reagent (Invitrogen, Carlsbad, CA) following the manufacturer's instructions. Quantity of the isolated RNAs was measured using NanoDrop 2000c spectrophotometer (ThermoFisher Scientific, Lenexa, KS) and the integrity of RNA was checked on 2% agarose gels. RNAs were reverse transcribed using Superscript III first strand cDNA synthesis kit (Invitrogen, Life Technologies) to specifically amplify *4*′*OMT2* (Isoform 2; GenBank: AY217334.1) and *7OMT* (GenBank: FJ156103.1) complementary DNA sequences.

### Construct preparation and transformation

Virus Induced Gene Silencing (VIGS) was carried out using TRV-based vector system. Fragments of 4′*OMT* (GenBank: AY217334.1) (293 bp) and *7OMT* (GenBank: FJ156103.1) (451 bp) were amplified with the primers listed in Supplementary Table 1. These primers, include regions of *BamhI* and *SmaI* restriction enzyme to be used in the next step. The amplified 4′*OMT* and *7OMT* genes were then inserted into pGEM-T vector (Promega, Madison, WI, USA). Both TRV and plasmid DNA were digested with *BamHI* and *SmaI* restriction endonucleases. The cleaved products were ligated into the TRV2 (individually pTRV2-4′*OMT* and pTRV2-7OMT) vectors. After cloning verification by sequencing, the constructs were separately transformed into *Agrobacterium tumefaciens* strain LBA4404 via electroporation. *A. tumefaciens* colonies separately transformed with TRV1 and pTRV2 with gene of interest were selected, then were grown in 10 mL of Luria-Bertani (LB) medium containing 50 mg/L kanamycin with overnight shaking at 250 rpm 28°C until cell absorbance reached to OD_600_ = 2. The cell cultures were centrifuged at 2000 g for 15 min, and the pellets were resuspended in 5 mL of induction buffer, (1 mM MES, 150 mM acetosyringone, and 10 mM MgCl_2_), pH:5.8, until OD_600_ reached to 0.8 for TRV2 and 0.2 for TRV1. Agrobacterium LBA4404 strains consisting TRV1 and TRV2 were then mixed in a 1:1 ratio. The mixture was used for agro-infiltration assay by needleless syringe to young leaves of opium poppy. The tip of the syringe without a needle suppressed against the underside of a leaf while synchronously applying kindly counterpressure to the other side of the leaf. Later, Agrobacterium is injected into the air space along the inside of the leaf stomata. The control plants were also agro-infiltrated with TRV2-Empty vector. The inoculation repeated two times with 7 days interval. One week after the second inoculation, the samples were harvested and stored at −80°C until use. The number of infiltrated plants was 15 for each pTRV2-4′*OMT* and pTRV2-7OMT experiment and among three of them which were almost equally silenced and the empty vector selected for further analyzes.

Over-expression assays were carried out that sequences containing full lengths of *4*′*OMT* and *7OMT* genes cloned into pGEM-T vector before cloned into a viral-based vector pGR106 containing 35S promoter. *4*′*OMT* (1077 bp) and *7OMT* (1170 bp) genes were amplified by PCR and ligated into pGEM-T vector. For this, forward primers containing *NotI* restriction site added the flag peptides (*4*′*OMT*-NotI FLAG and 7OMT-NotI FLAG) and reverse primers (4-OMT-SalI R and *7-OMT*-SalI R) were used for cloning (Supplementary Table 2). Following the confirmation of clonings by sequencing, the constructs were transformed into pGR106 viral-based vector. Subsequently, the constructs were transformed into LBA4404 strain of *A. tumefaciens* by electroporation. *A. tumefaciens* colonies with pGR106-*4*′*OMT* and pGR106-7OMT constructs were then grown in 10 mL of LB medium containing 50 mg/L kanamycin overnight shaking at 250 rpm 28°C until OD_600_ reached to 2.

The pelleted cultures were dissolved in an induction buffer (pH:5.6) containing 1 mM MES, 150 mM acetosyringone, and 10 mM MgCl_2_ until the OD_600_ reached to 0.4, and were further incubated overnight at room temperature. The agro-infiltration was applied on capsule, leaf and stem tissues. The mock control plants were also agro-infiltrated with pGR106-Empty vector 10 days after inoculation, samples were harvested and stored at −80°C until use.

### Quantitative RT-PCR analysis

To identify expression levels of *4*′*OMT* and *7OMT* in overexpressed and silenced tissues, qRT-PCR assays were performed using LightCycler 480 Real-Time PCR System (Roche, Mannheim, Germany). Additionally, the transcript levels of some BIA biosynthesis genes such as *COR, SAT, TYDC, CNMT, 6-OMT* were also measured. The primers used for measurements were given in Supplementary Table 3. The qRT-PCR experiments were performed as the following; cDNA (2 μl) was amplified with 10 mM of each specific forward and reverse primers and 10 μl SYBR Green I Master mix (Roche Applied Science, Penzberg, Germany) in a total volume of 20 μl. PCR amplification was generated as follows; preheating at 95°C for 5 min; and 41 cycles of 95°C for 10 s, 55–60°C for 20 s, and an extension at 72°C for 10 s. Three biological replicates were performed for each sample. qRT-PCR experiments were performed in triple replicates for each RNA sample/primer combinations. Gene expression levels were calculated according to the 2^−Δ*ΔCt*^ method (Livak and Schmittgen, 2001). To normalize the results, *18S* rRNA gene, housekeeping gene was used as reference gene (Supplementary Table 3). The melting curves templates for qRT-PCR were carried out from 57 to 95°C, as the temperature increased at 0.5°C per second. As a result of the melting curves were analyzed for each run and the data of the fluorescence signals, which filter out the false-positive peaks.

### Metabolite measurement

Plant materials were dried at 28°C for 2 days. Dried samples (0.1 g) were soaked in methanol (HPLC grade, Merck, German) at room temperature for 1 day with shaking followed by filtration to separate the marc and evaporate. The extracts were solved in 2000 ppm and diluted to 1/200 ppm from stock. To analyze the HPLC-TOF/MS, the samples were filtered through 0.45 μm membranes. Morphine, codeine, thebaine, papaverine, noscapine, and laudanosine were analyzed using specific standards. Alkaloids were quantitated in Agilent 1260 Infinity HPLC system (Agilent, Palo Alto, CA) coupled with Agilent 6210 TOF-MS detector and Agilent EC 250/4 Nucleosil 100–5 (HPLC Column, Nucleosil C18, 100A, 5 μm, 4 × 250 mm). The column temperature was adjusted at 35°C and the injection volume was set to 10 μL. Mobile phases A and B were water/1 mL L^−1^ acetonitrile (0.1% formic acid), respectively. The elution program was: 0–6 min, 40% B; 6–10 min, 50% B; 10–15 min, 90% B; 15–16 min, 90% B; 16–25 min, 40% B. All the measurements for each sample were triplicated.

## Results

In this study, we have successfully manipulated *4*′*OMT* and *7OMT* genes in capsule, stem, and leaf tissues of opium poppy. Expression profiles of mRNAs, and alkaloids were analyzed accordingly using proper approaches. We observed correlated results between silenced and overexpressed tissues.

### Silencing of *4′OMT*

**Figure 2:**
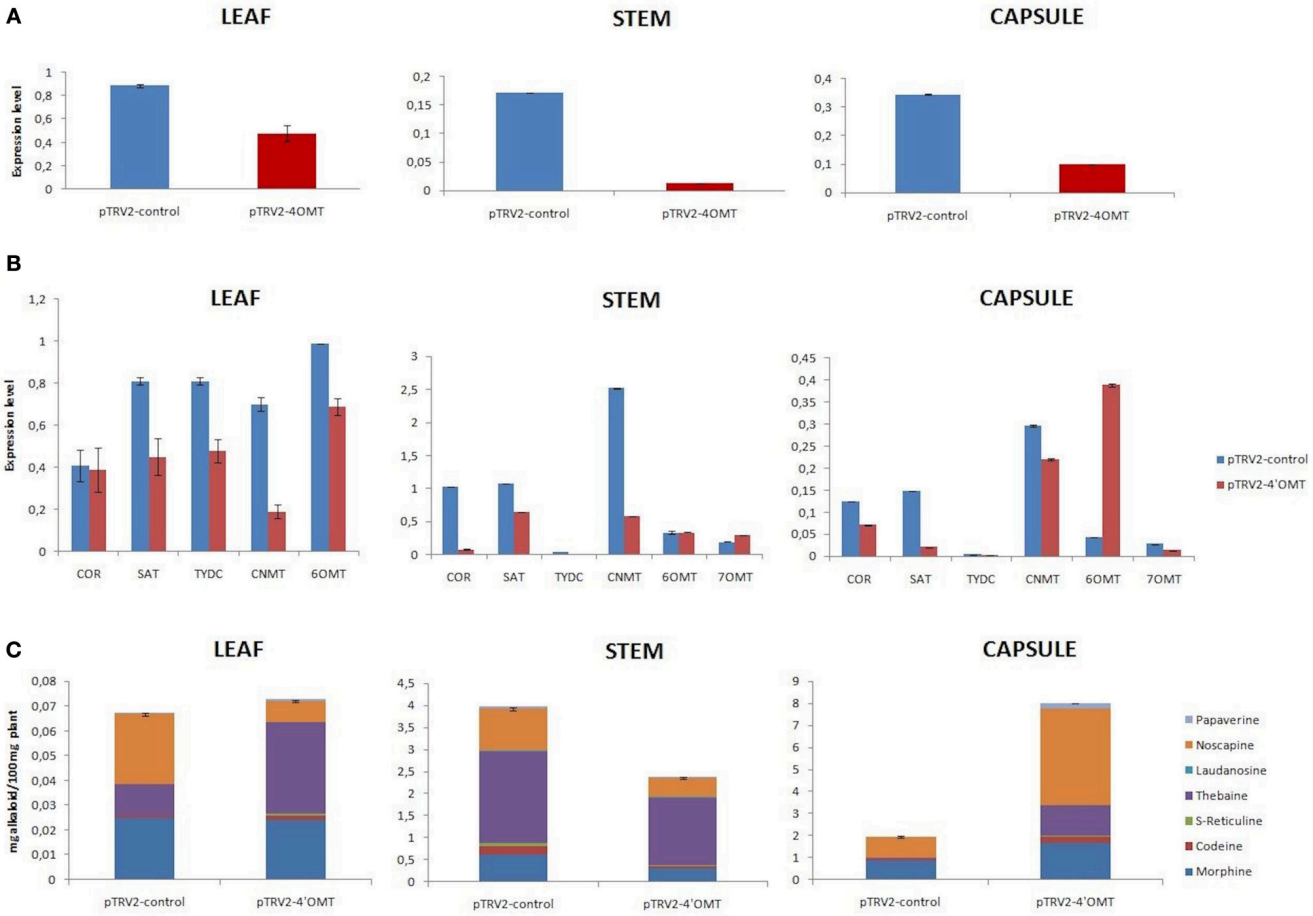
*****4***′***OMT*** gene silencing results in different tissues (leaf, stem, and capsule) of opium poppy**. **(A)** *4*′*OMT* gene silencing level measurements via qRT-PCR. **(B)** Expression level analysis of BIA biosynthesis genes upon of 4′*OMT* VIGS by qRT-PCR. **(C)** Measurement of alkaloids in *4*′*OMT* silenced and control opium poppy plants.

Effective silencing on *4*′*OMT* gene was obtained via agro-infiltration upon VIGS in opium poppy with the rates of 92, 71, and 46% in stem, capsule, and leaf tissue, respectively, (Figure 2A). To measure the effect of *4*′*OMT* suppression on the other selected transcripts involved in BIA biosynthesis, qRT-PCR assay was carried out and the results showed that these genes were differentially expressed in different tissues. With the exception of the expression level induction in *7OMT* and *6OMT* genes, all measured genes were detected as down-regulated in stem (Figure 2B). The lowest expression was obtained in *COR* transcript level taking part in downstream of morphinan branch upon *4*′*OMT* silencing in the stem. On the other hand, *SAT* expression level was strongly reduced in the capsule compared to other transcripts via *4*′*OMT* silencing. Although suppression in BIA biosynthesis genes was measured in the capsule, *6OMT* expression was detected as up-regulated. In leaves, the silencing of *4*′*OMT* caused suppression of BIA gene expressions. Among them, the expression of *CNMT* and *6OMT* was highly down-regulated with a rate of 72 and 70%, respectively, (Figure 2B). Silencing of *4*′*OMT* resulted in differential accumulation of BIAs in different tissues. Morphine, codeine, s-reticuline, papaverine, noscapine, thebaine, and laudanosine levels were measured via HPLC-TOF/MS upon *4*′*OMT* VIGS. First of all, the relative alkaloid abundance in silenced stem tissue was 41% lower than the control plants (Figures 2C, **6A**; Supplementary Figure 1). Substantial reduction was detected in the accumulation of most of the measured alkaloids upon *4*′*OMT* silencing in stem tissue. One the other hand, a considerable induction of total alkaloid content was detected in the capsule. Higher accumulation of morphine, codeine, thebaine, and noscapine rather than papaverine and laudanosine was observed in silenced capsule. In *4*′*OMT*-silenced leaf, no significant change was observed in total alkaloid content. Despite comparable total alkaloid amount measured between silenced and control leaf samples, levels of noscapine, and thebaine were changed inversely (Figures 2C, **6A**).

### *7OMT* gene silencing

**Figure 3:**
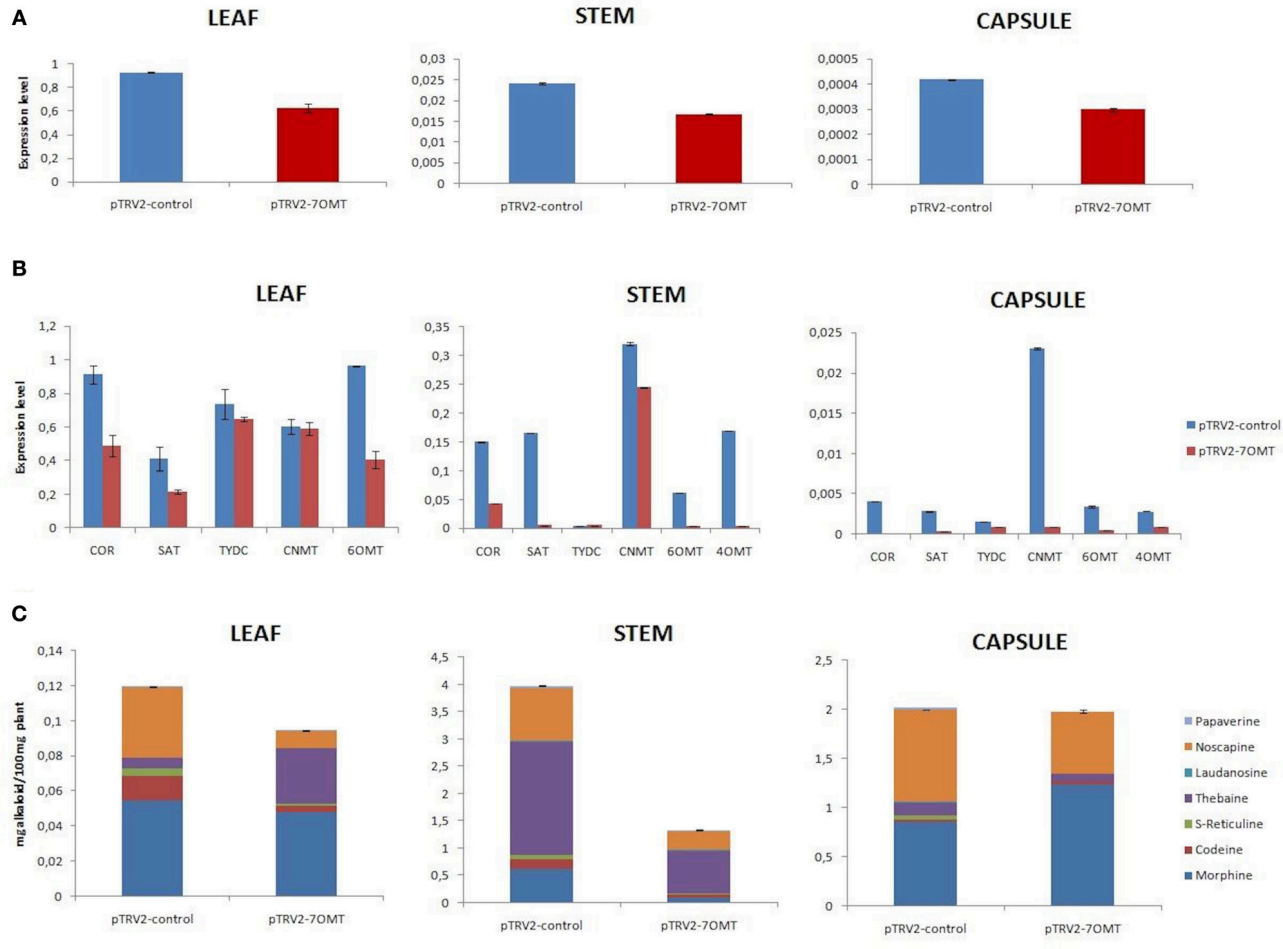
*****7OMT*** gene silencing results in different tissues (leaf, stem, and capsule) of opium poppy. (A)** *7OMT* gene silencing level measurements via qRT-PCR. **(B)** Expression level analysis of BIA biosynthesis genes upon of 7OMT VIGS by qRT-PCR. **(C)** Measurement of alkaloids in *7OMT* silenced and control opium poppy plants.

VIGS assay was also conducted to discover functional role of *7OMT* gene in BIA biosynthesis. *7OMT* transcript was successfully suppressed in stem, capsule, and leaves with the rate of 30, 28, and 32%, respectively, (Figure 3A). Upon silencing, expression levels of selected BIA biosynthesis genes were differentiated in opium poppy tissues (Figure 3B). It was detected that expression levels of *6OMT, 4*′*OMT, SAT*, and *COR* genes were highly reduced upon *7OMT* gene silencing in stem. Nevertheless, expression of *TYDC* gene was found to be induced. In addition transcript levels of measured genes were reduced in capsule tissue. Among them, strong reduction was detected in *COR, CNMT*, and *SAT* genes. Furthermore, *7OMT* VIGS caused suppression of gene expressions involved in BIA biosynthesis in leaf tissue. *7OMT* gene silencing resulted in 66% reduction of total alkaloid accumulation in stem. Amount of the each measured alkaloid was reduced upon *7OMT* VIGS in stem (Figures 3C, **6A**). Among them, morphine content was remarkably suppressed approximately six-fold, whereas reduction of other alkaloids was about three-fold. In opposition, *7OMT* VIGS in capsule caused induction of morphine levels. Meanwhile, no significant change was observed in total alkaloid accumulation. On the other hand, compared to morphine level, approximately 1.5-fold lower accumulation of thebaine and noscapine was measured in capsule. Upon *7OMT* VIGS, it was observed that noscapine and codeine amounts were significantly reduced in leaf tissue (Figures 3C, **6A**).

### Transient overexpression of *4′OMT* gene in opium poppy

**Figure 4:**
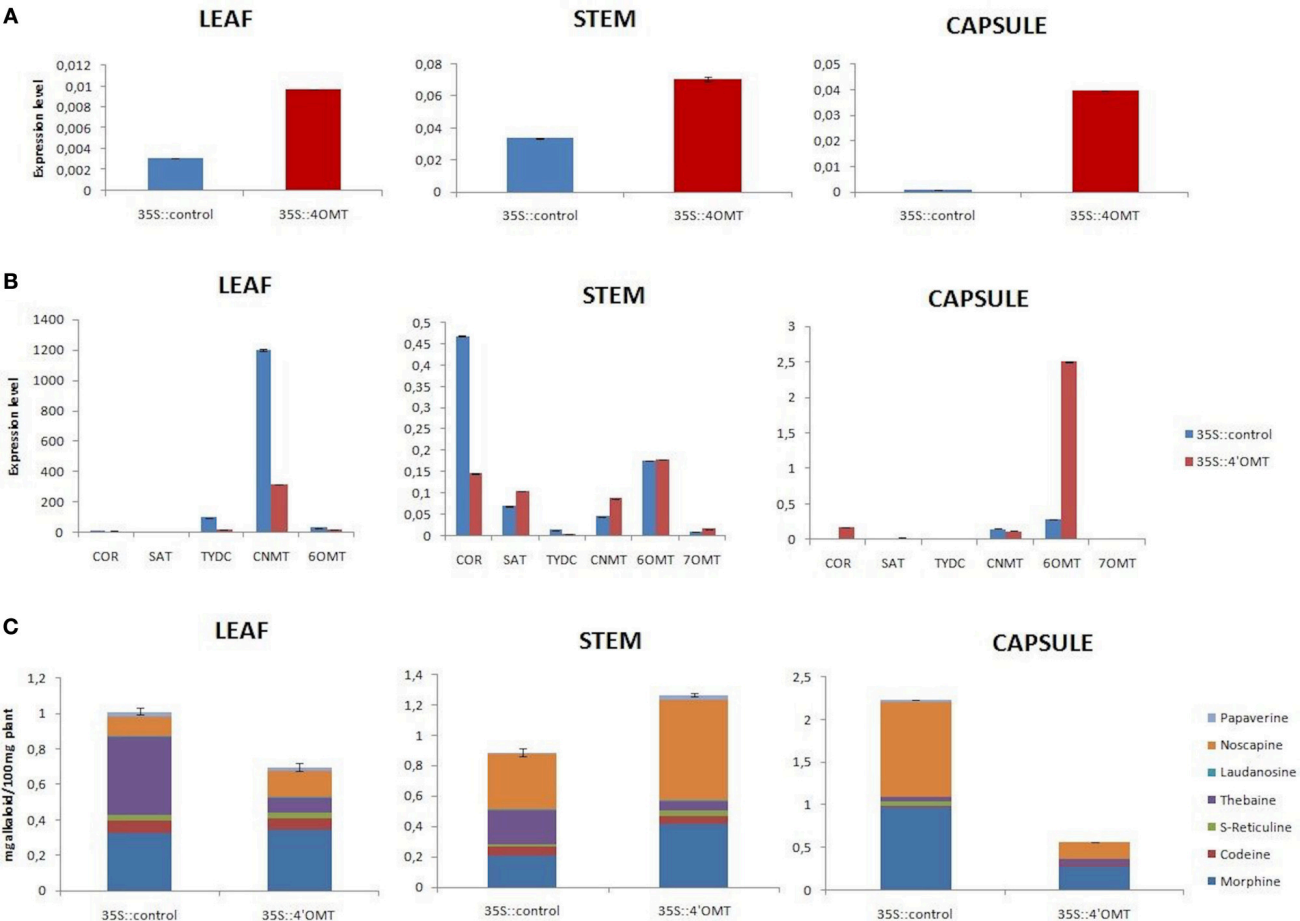
*****4***′***OMT*** overexpression results in different tissues (leaf, stem, and capsule) of opium poppy**. **(A)** Measurements of *4*′*OMT* overexpression level via qRT-PCR. **(B)** Expression level analysis of BIA biosynthesis genes upon of 4′*OMT* overexpression by qRT-PCR. **(C)** Measurement of alkaloids in *4*′*OMT* overexpressed and control opium poppy plants.

*4*′*OMT* gene was successfully overexpressed under the viral constitutive CaMV35S promoter. The highest overexpression level was found to be 475% in capsule (Figure 4A). According to the expression measurement assay results, the most effected transcript was *6OMT* in capsule. Moreover, the overexpression of *4*′ *OMT* caused a significant increase in the expression of *CNMT, SAT*, and *7OMT* transcript levels in stem, while a negative regulation was observed for *COR* gene. It was measured that *4*′*OMT* was strongly expressed in leaf tissue with a rate of 210% which caused down-regulation of *CNMT, TYDC*, and *6OMT* gene expressions (Figure 4B). The highest induction rate was 109% in stem tissue. This overexpression led to induce the *SAT* and *CNMT* transcripts. Alkaloid profiles were also affected by the overexpression of *4*′*OMT* in opium poppy tissues (Figures 4C, **6A**). In stem, increased amount of (S)-reticuline was observed which caused 43% induction of total alkaloid content. Moreover, it was detected that both morphine and noscapine levels induced about two-fold. Total alkaloid measurement in leaf showed that any significant difference was present upon the overexpression. There was a minor alteration for noscapine content in overexpressed opium poppy. On the other hand, a considerable suppression of total alkaloid content (75%) was measured in capsule in response to *4*′*OMT* overexpression. The highest reduction was detected for morphine and noscapine content (Figures 4C, **6A**).

### *7OMT* overexpression in opium poppy

**Figure 5:**
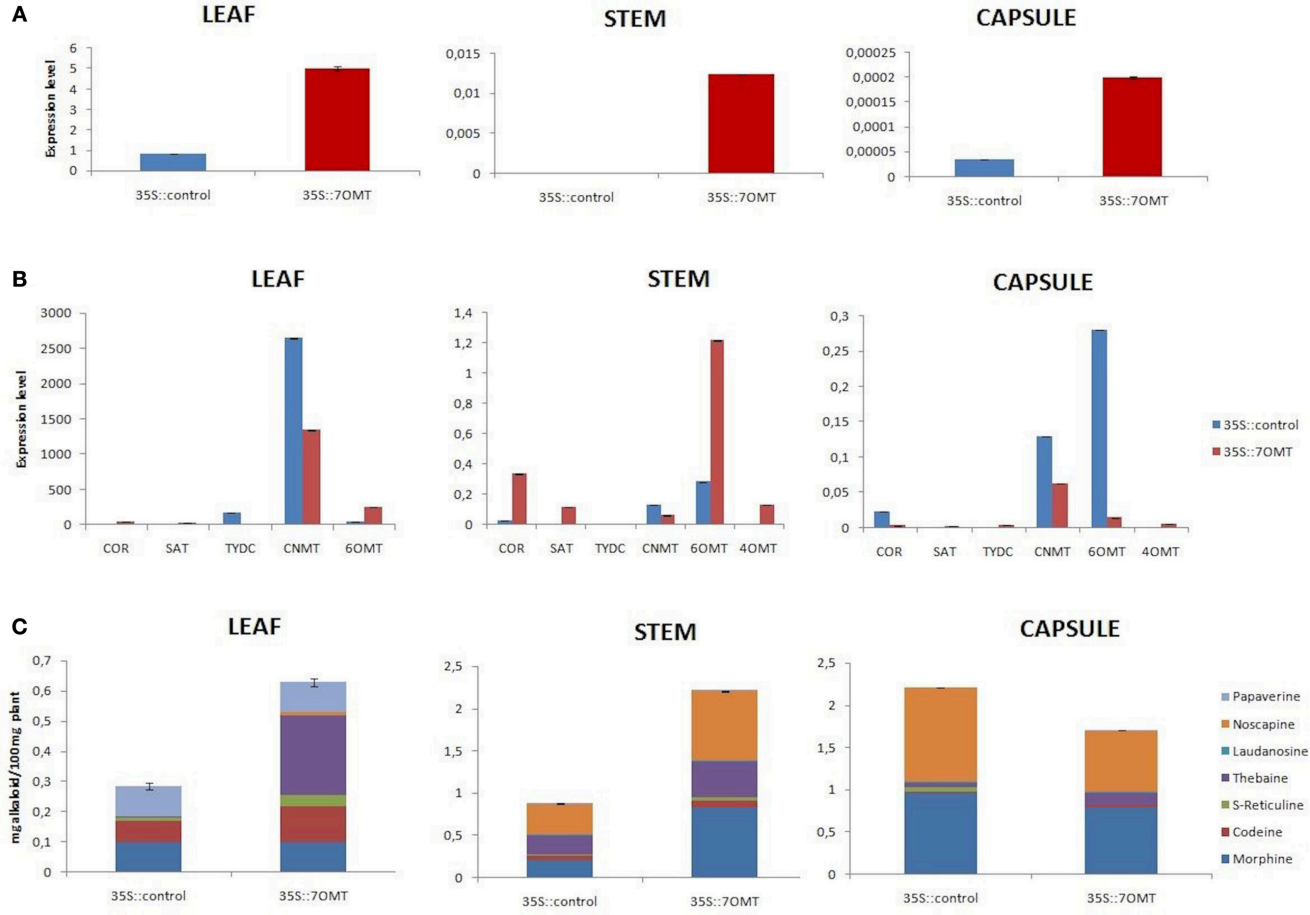
*****7OMT*** overexpression results in different tissues (leaf, stem, and capsule) of opium poppy**. **(A)** Measurements of *7OMT* overexpression level via qRT-PCR. **(B)** Expression level analysis of BIA biosynthesis genes upon of 7OMT overexpression by qRT-PCR. **(C)** Measurement of alkaloids in *7OMT* overexpressed and control opium poppy plants.

**Figure 6:**
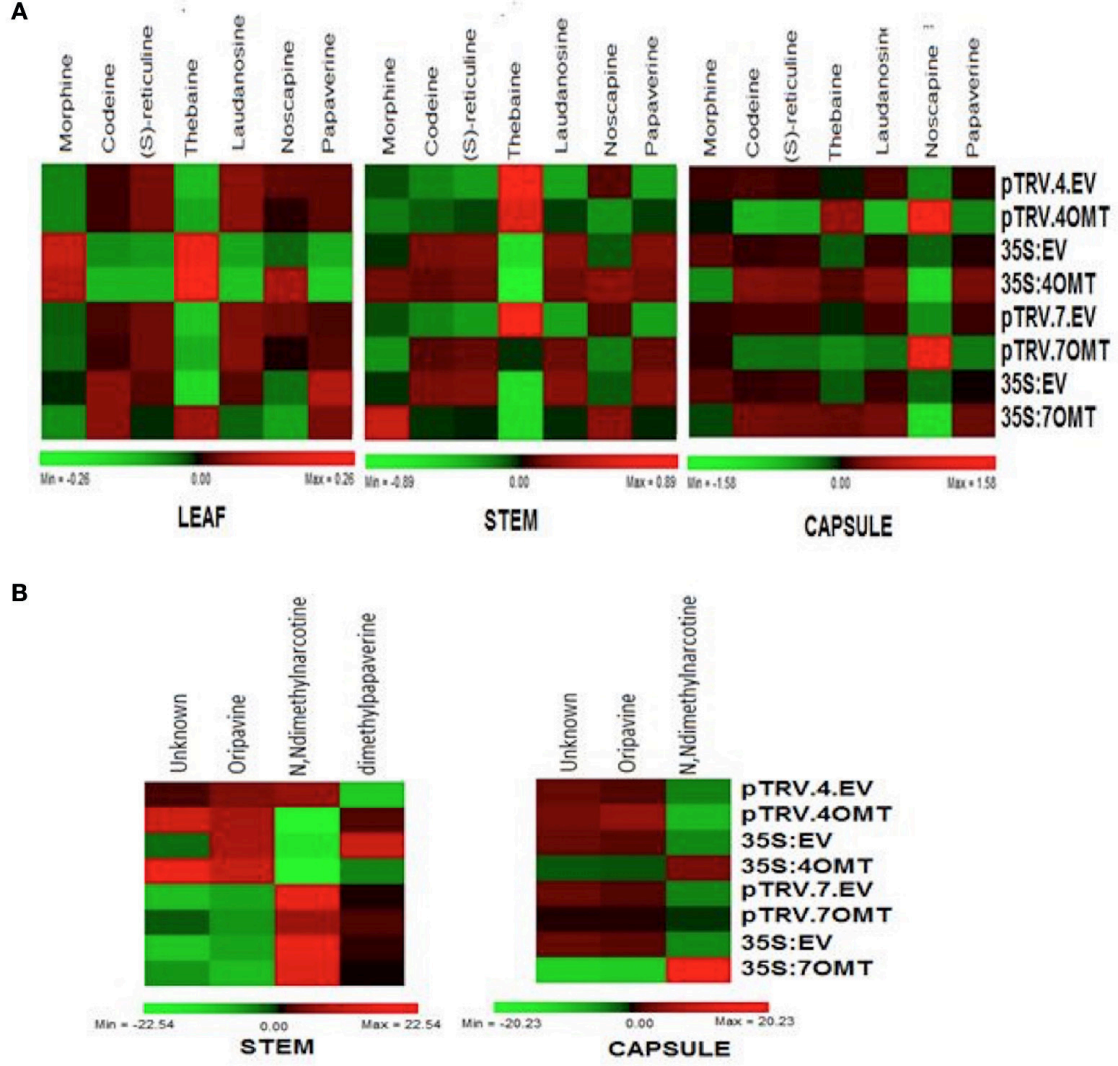
**(A)** Heatmap representing the abundance of morphine (M), codeine (C), S-reticuline (R), thebaine (T), laudanosine (L), noscapine (N), and papaverine (P) for VIGS and overexpression of both 4′*OMT* and 7OMT in leaf, stem, and capsule in opium poppy. **(B)** Heatmap showing the relative alterations in unknown metabolites (U) or oripavine (O), N, Ndimethylnarcotine (A), and dihydropapaverine (D) upon silencing and overexpression applications in stem and capsule.

Here, we performed overexpression of *7OMT* gene in all target tissues of opium poppy. Overexpression assay revealed induction of *7OMT* in leaf, stem, and capsule with 497, 7614, and 471%, respectively, (Figure 5A). The levels of *COR, SAT, 6OMT*, and *4*′*OMT* gene transcripts increased considerably in stem. However, *CNMT, 6OMT*, and *COR* levels were significantly decreased in capsule (Figure 5B). The results obtained from leaf tissue demonstrated an increase of *6OMT* and decrease of *CNMT* and *TYDC* expressions. Overexpression of *7OMT* gene led to down-regulation of *CNMT* in all tissues (Figure 5B). A reverse expression pattern of *6OMT* was observed between stem and capsule tissues. Its expression was induced in stem while a down-regulation was detected in capsule. The over-expression of *7OMT* resulted in a considerable increase of alkaloid accumulation in stem and leaf tissues. Furthermore, higher morphine concentration was measured in stem compared to control plant. Additionally, overexpression of *7OMT* reduced noscapine level remarkably in capsule (Figures 5C, 6A).

In addition to major BIAs, we also measured other types of alkaloids such as oripavine, N, N-dimethylnarcotine, dimethylpapaverine, and unidentified molecules in capsule and stem tissues (Figure 6B). N, N-dimethylnarcotine level was reduced while dimethylpapaverine level was induced in stem. However, any significant change was detected in capsule upon *4*′*OMT* silencing (Figure 6B). On the other hand, *7OMT* overexpression caused increase of N, N-dimethylnarcotine in capsule.

## Discussion

Plants are being used as source of secondary metabolites for medicinal purposes. Opium poppy, an agronomically and medicinally important crop, produces opiates such as morphine and noscapine (Unver et al., 2010). In order to understand the biosynthesis mechanism of alkaloids, new approaches such as the examination of the EST database, microarray screening, metabolic engineering, and proteomic tools were applied (Takemura et al., 2013; Gurkok et al., 2015; Ramegowda et al., 2014; Schütz et al., 2014). Furthermore, viral-based gene silencing and over-expression studies were utilized for functional analysis of genes involved in BIA biosynthesis in opium poppy (Allen et al., 2008; Wijekoon and Facchini, 2012; Dang and Facchini, 2014).

Here, to functionally analyze the regulatory roles in BIA biosynthesis in tissue specific manner, expression levels of *4*′*OMT* and *7OMT* were successfully manipulated in opium poppy. To date, while VIGS of *4*′*OMT* and *7OMT* was reported in opium poppy (Desgagné-Penix and Facchini, 2012) overexpression results of these genes have not been presented yet.

Silencing and overexpression of genes involved in BIA biosynthesis led to the accumulation of an altered amount of alkaloids in opium poppy tissues (Figures 2C, 3C, 4C, 5C). Moreover, transcript levels of BIA biosynthetic genes were differentially expressed within targeted tissues (Figures 2B, 3B, 4B, 5B). Similarly, Dang and Facchini (2014), showed that VIGS-mediated suppression of *CYP82Y1* gene in opium poppy resulted in diverse transcript levels in different opium poppy tissues (Dang and Facchini, 2014). In another study, the BIA synthetic gene expression differentiation was also reported upon systematic silencing of morphinan pathway genes (Wijekoon and Facchini, 2012).

*4*′*OMT2* gene silencing caused reduction in all measured alkaloids in the stem and that total alkaloid content decreased by 41% (Figure 2C). Similarly, Desgagné-Penix and Facchini (2012) also reported that the silencing of *4*′*OMT2* in the stem resulted in a 43% total alkaloid reduction (Desgagné-Penix and Facchini, 2012). On the other hand, the total alkaloid accumulation was induced by 43% via overexpression of *4*′*OMT* in the stem. Though, Inui et al. (2007) measured the total alkaloid level change upon *4*′*OMT* overexpression in *Coptis japonica* (Inui et al., 2007). In *C. japonica* the overexpression of this gene had a lesser extent on total alkaloid accumulation. The inconsistency between our results and *C. japonica* overexpression outcomes might have resulted from the different metabolic branches of BIA pathway in host plants.

We detected correlating morphine content alteration upon gene silencing and overexpression. The silencing of *4*′*OMT* caused approximately a two-fold reduction whereas overexpression resulted in a two-fold induction of morphine content in stem (Figures 2C, 4C). Therefore, manipulation of *4*′*OMT* leads consistent regulation on morphine content. On the other hand, the expression level differentiation of *CNMT* and *SAT* was found to be consistent with the manipulation of *4*′*OMT*. Therefore, *4*′*OMT* might positively regulate the expression of these genes by adjusting the morphine accumulation (Figures 2, 4). Likewise, recently it was reported that *4*′*OMT* expression might be rate-limiting on BIA biosynthesis (Desgagné-Penix and Facchini, 2012). Frick et al. (2007) discussed the rate-limiting activity of *NMCH* gene for BIA accumulation in opium poppy. It could be concluded that BIA biosynthesis rate-limiting regulation does not depend on only one gene (Frick et al., 2007).

Different alkaloid profiles were analyzed in a capsule tissue from that of a leaf and stem by manipulation of *4*′*OMT* gene. The accumulation of morphine, thebaine, and noscapine were increased in the capsule tissue upon *4*′*OMT* VIGS. On the other hand, their production was suppressed by *4*′*OMT* over expression. Though the expression level of *COR* and *SAT* genes were found to be down-regulated, in a capsule of *4*′*OMT* they were silenced. It was previously stated that overexpression of *COR* and *SAT* genes caused total alkaloid accumulation in opium poppy (Allen et al., 2004, 2008; Larkin et al., 2007). Therefore, morphine accumulation in a capsule might be explained by the induction of *COR* and *SAT* gene in the stem and then the transportation of intermediate products from stem to capsule tissue. Furthermore, it can be speculated that an unidentified pathway might have resulted in the accumulation of more alkaloids in the capsule.

Up to date, it has been reported that silencing of *7OMT* regulates laudanosine biosynthesis (Desgagné-Penix and Facchini, 2012). Here, we showed for the first time that *7OMT* has also considerable effect on biosynthesis of morphine, thebaine and noscapine. Due to different roles of stem and capsule tissues for BIA biosynthesis and accumulation, *7OMT* gene might be involved in the biosynthesis of morphine at tissue specific manner. Manipulation of *7OMT* altered the amount of morphine in stem. A similar pattern was observed for thebaine accumulation in stem by *7OMT* manipulation (Figures 3C, 5C). Our results are consistent with the fact that morphine and thebaine alkaloids are in the same branch of BIA pathway, thus *7OMT* shows similar impact on biosynthesis of these alkaloids. Surprisingly, we showed the regulatory action of *7OMT* on noscapine biosynthesis, which is placed on a different branch then morphinan alkaloids. Moreover, opposite levels of noscapine were measured between stem and capsule upon manipulation of *7OMT* implying tissue specific regulation of *7OMT*. Manipulation of *7OMT* has impact on expression of *6OMT* at different levels in tissues (Figures 3B, 5B). In stem, upon *7OMT* VIGS we observed the suppression of *6OMT* expression and reduction in total alkaloid amount (Figures 3B,C). Similarly, Desgagné-Penix and Facchini (2012) found decrease in total alkaloid content upon *6OMT* VIGS (Desgagné-Penix and Facchini, 2012). However, a different pattern was observed in *7OMT*-silenced capsule tissue supporting tissue specific action of *7OMT*. Since the whole BIA pathway is still unclear, these outcomes might be sourced by putative unknown pathways. As discussed by Leonard et al. (2009), metabolic engineering approaches such as over-expression of a gene can lead to the accumulation of unexpected alkaloid product because of the complex pathways (Leonard et al., 2009).

An unexpected correlation between *COR* transcript level and thebaine accumulation in stem for both *4*′*OMT* and *7OMT* assays suggests that the *COR* encoding gene may have an important effect on the biosynthesis of thebaine. The results showed that where the *COR* down-regulated thebaine content was decreased or the up-regulation of *COR* induced the thebaine levels in stem and capsule with the exception *7OMT* induced capsule. Consistent with our results, the silencing of *COR* caused a decrease in salutaridine, thebaine, codeine, morphine in latex (Wijekoon and Facchini, 2012). Additionally, RNAi applications of *COR* and *SAT* genes altered the alkaloid levels of intermediates (Allen et al., 2004; Kempe et al., 2009; Wijekoon and Facchini, 2012). Grothe et al. (2001) showed that the presence of *SAT* transcript leads to the accumulation of morphinan alkaloids such as morphine, thebaine, and codeine. *SAT* transcripts levels in stem revealed a correlation especially with morphine accumulation for both suppression and over-expression assays but the same similarity could not be found for other alkaloids. Therefore, both *4*′*OMT* and *7OMT* might have an effect on *SAT* transcript in biosynthesis of morphine.

## Conclusion

In the presented study, through the metabolic engineering approaches, we present new results about the regulatory roles for *4*′*OMT* and *7OMT* in BIA biosynthesis. Upon silencing and overexpression applications, tissue specific effects of these genes were identified. Manipulation of *4*′*OMT* and *7OMT* genes caused differentiated accumulation of BIAs including morphine and noscapine in capsule and stem tissues.

## Author contributions

TG, MT, and TU drafted the paper, MT and TU analyzed the data, TU, IP, and SÖ organized and materials and planned the study. EO and TU performed experiments. ID conducted the metabolite measurements. Aİ and SO helped plant growth and inoculation assays.

### Conflict of interest statement

The authors declare that the research was conducted in the absence of any commercial or financial relationships that could be construed as a potential conflict of interest.
