# Supplementary material for: Functional Characterization of 4′OMT and 7OMT Genes in BIA Biosynthesis
Source: Front Plant Sci. 2016 Feb 16;7:98. doi: 10.3389/fpls.2016.00098 (PMC4754624; doi:10.3389/fpls.2016.00098)
Supplement: Supplementary file 1 [file Table1.DOCX]

**Supplementary Table 1.** The primers were used to clone of *4´OMT* and *7OMT* genes in silencing experiment.

| Primer names | Primer sequence (5'->3') |
| --- | --- |
| 4OMT-BamHI (Forward)  4OMT-SmaI (Reverse) | GCGGGATCCTAGCACTTGAAGAAGAATCCA  GCGCCCGGGAGGAAATAGGAAACCATTTCC |
| 7OMT-BamHI (Forward)  7OMT-SmaI (Reverse) | GCGGGATCCAACATCCTTCACGATTGGAAC  GCGCCCGGGCCAAACATAATACATCAGATT |
