# Supplementary material for: Functional Characterization of 4′OMT and 7OMT Genes in BIA Biosynthesis
Source: Front Plant Sci. 2016 Feb 16;7:98. doi: 10.3389/fpls.2016.00098 (PMC4754624; doi:10.3389/fpls.2016.00098)
Supplement: Supplementary file 2 [file Table2.DOCX]

**Supplementary Table 2.** The primers were performed to clone of *4´OMT* and *7OMT* genes in over expression experiment.

| Primer names | Primer sequence (5'->3') |
| --- | --- |
| 4´OMT-NotI FLAG (Forward)  4-OMT-SalI (Reverse) | GGAGCGGCCGCATGGATTACAAGGATGACGACGATAAGatgggtagtttagatgcaaaacc  *GGAGCgtcgac*TACTAAGGGAAGGCTTCAATG |
| 7OMT-NotI FLAG (Forward)  *7-OMT*-SalI (Reverse) | GGAGCGGCCGCATGGATTACAAGGATGACGACGATAAGatggaagtagttagccagattg  *GGAGCgtcgac*CTAACAAAGTCTCGCCCTCCA |
