# Supplementary material for: Functional Characterization of 4′OMT and 7OMT Genes in BIA Biosynthesis
Source: Front Plant Sci. 2016 Feb 16;7:98. doi: 10.3389/fpls.2016.00098 (PMC4754624; doi:10.3389/fpls.2016.00098)
Supplement: Supplementary file 3 [file Table3.DOCX]

**Supplementary Table 3.** The primers used for qRT-PCR analysis.

| **Gene Bank** | **Description** | | | **Forward Primer (5'->3')** | | **Reverse Primer (5'-3')** |
| --- | --- | --- | --- | --- | --- | --- |
| X16077.1 | | 18S rRNA | TTTGACTCAACACGGGGAAA | | CAGACAAATCGCTCCACCAA | |
| FJ624147.1 | | COR | TGATCTTGTCCTCCCTGCTC | | TCAAGCTTACCGGATGGTGT | |
| FJ200354.1 | | SAT | CAACAAGGGTTGAGGTGGTT | | TGGTGGGTCCATTTTCTTTC | |
| AF025435.1 | | TYDC | GCAACCTCCAAGGCTA CAAA | | TGGCACAGAGAAACAATGGA | |
| DQ028579.1 | | CNMT | TCAG CAACAATAGACGAAGCA | | AGACCACCTTGACCACAACC | |
| AY268893.1 | | 6-OMT | CACGACTGGAGTGACGAAGA | | TCGCAACACACA ATCAACAA | |
