# Supplementary material for: Functional Characterization of 4′OMT and 7OMT Genes in BIA Biosynthesis
Source: Front Plant Sci. 2016 Feb 16;7:98. doi: 10.3389/fpls.2016.00098 (PMC4754624; doi:10.3389/fpls.2016.00098)
Supplement: Supplementary file 4 [file DataSheet1.DOC]

**Figure 1:**HPLC-TOF analysis indicated the relative abundance of major benzylisoquinoline alkaloids of inoculated and empty vector(EV) control plants.


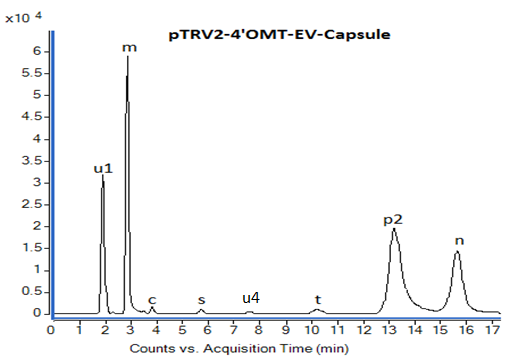

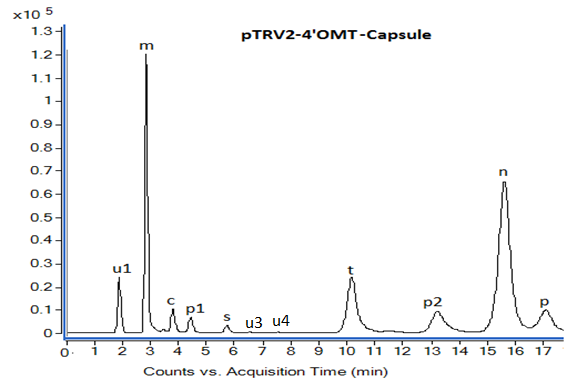


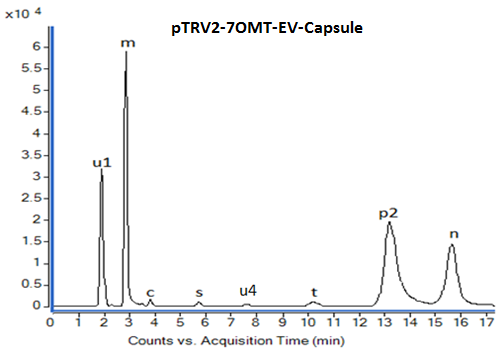

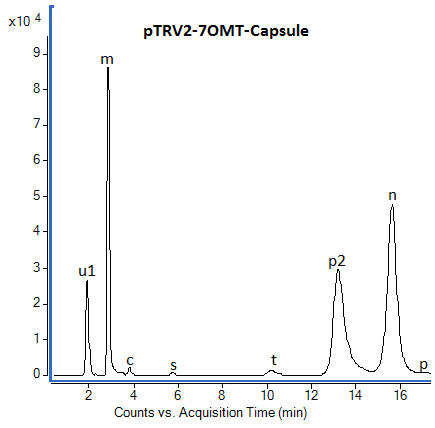


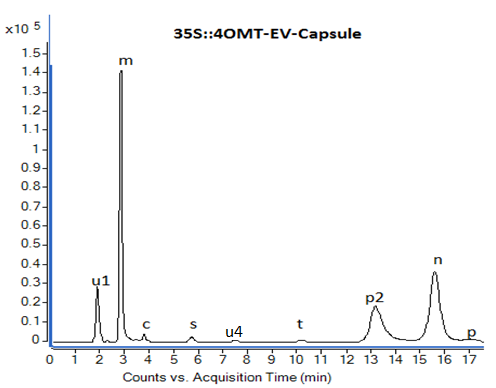

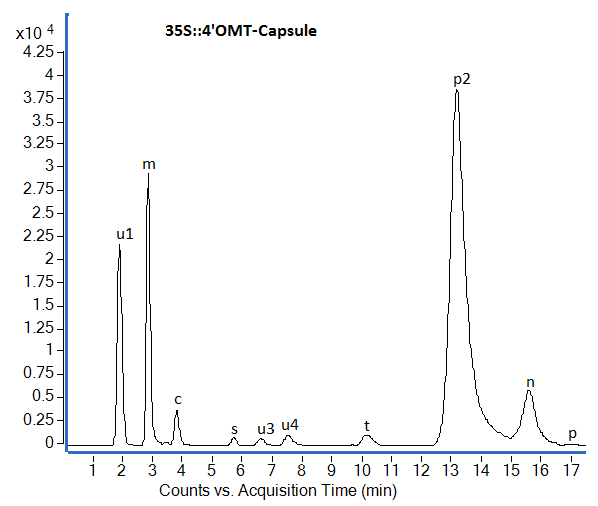


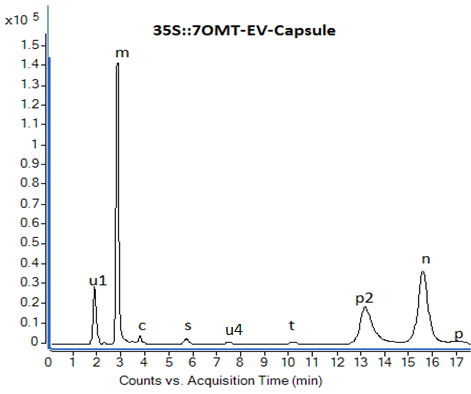

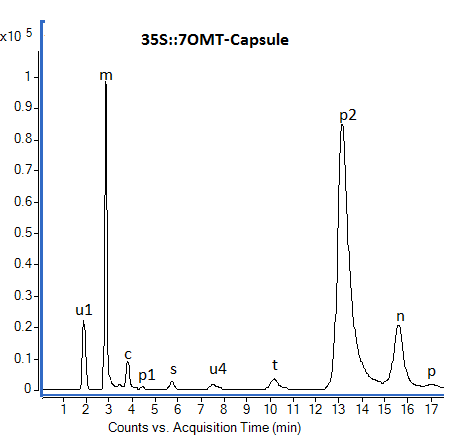


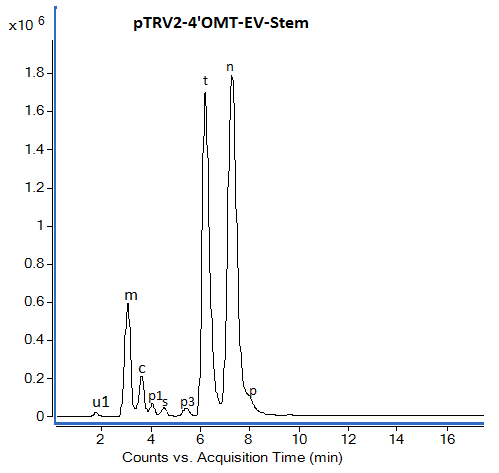

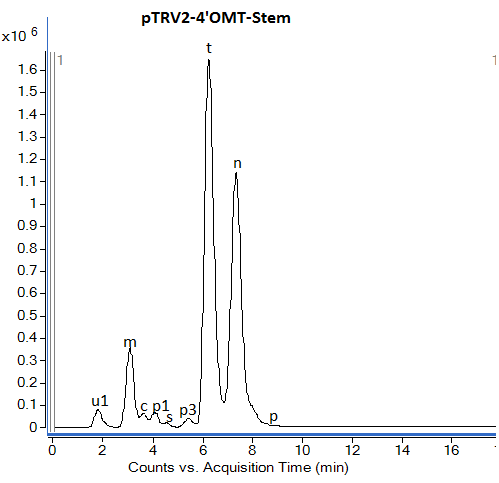


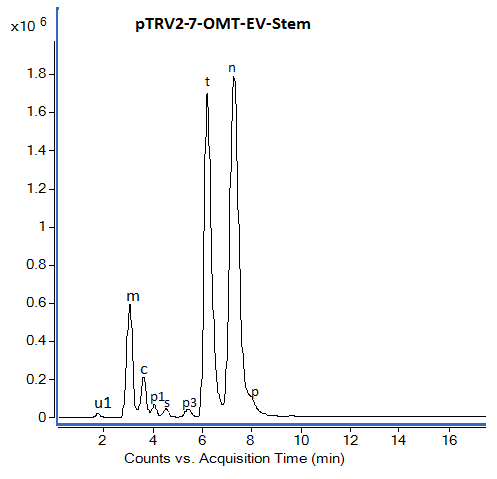

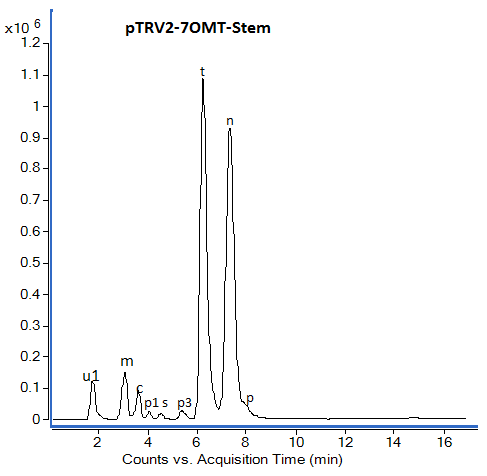


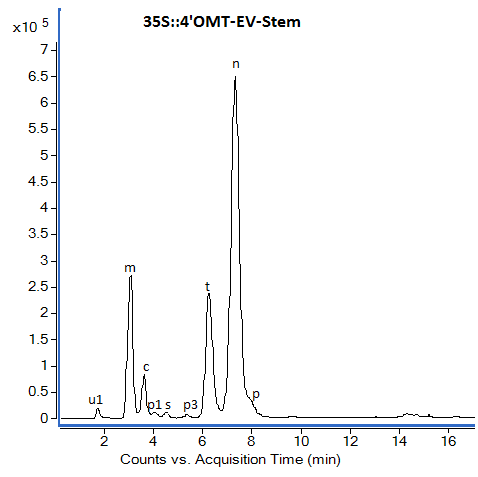

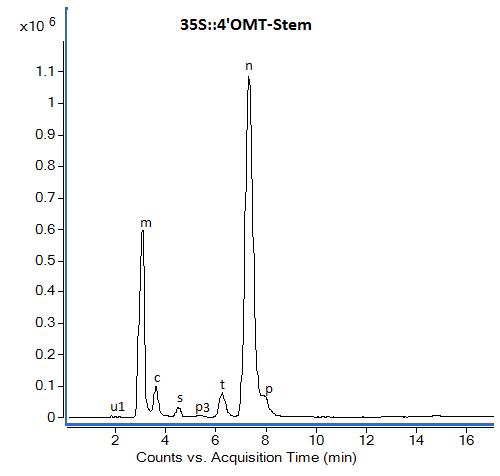


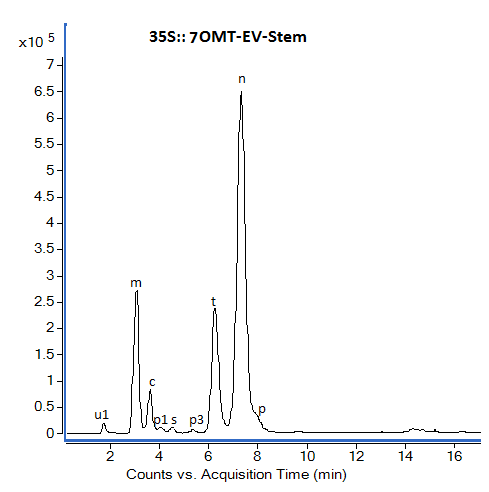

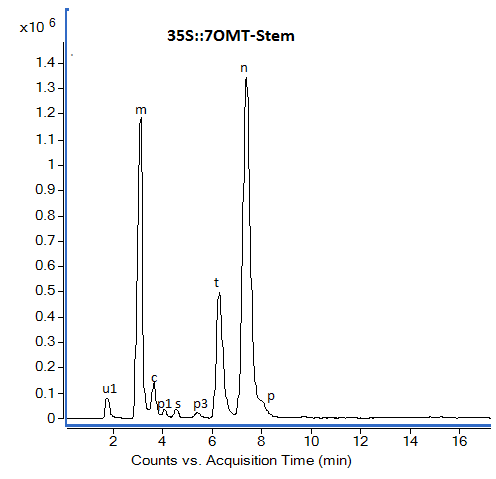


Abbreviations: m, morphine; c, codeine; s, salutaridine; r, reticuline; t, thebaine; n, noscapine; p, papaverine; u1, unknown 1; u2, unknown 2; u3, unknown 3; u4, unknown 4; p1, predictedoripavine; p2, predicted N’N dimetylnarcotine; p3, dihydropapaverine.
